# AMN Congress 2022 – Report of the panel on the effective treatment solutions for post-TBI cognitive problems

**DOI:** 10.25122/jml-2022-1012

**Published:** 2022-07

**Authors:** Alexandra-Mihaela Gherman, Andreea Strilciuc, Dafin Fior Muresanu

**Affiliations:** 1. RoNeuro Institute for Neurological Research and Diagnostic, Cluj-Napoca, Romania; 2. Department of Neuroscience, Iuliu Hatieganu University of Medicine and Pharmacy, Cluj-Napoca, Romania

"The incidence of post – TBI cognitive problems is known – what should be done to offer effective treatment solutions?" – this is the question the panelists

- Nada Andelic (Norway)
- Juan Carlos Arrago Lasprilla (Spain)
- Peter Lackner (Austria)
- Katrin Rauen (Switzerland)
- Nicole von Steinbüchel (Germany)
- Marina Zeldovich (Germany)

offered answers to, definitely placing their statements in the extremely dynamic, digitally developed world context.

It is Prof. Dafin F. Muresanu who provided the start point of the dialogue by suggesting a potential future medical context with precisely defined tools to identify those who will develop a patterned cognitive impairment from the population with mild cognitive impairment.

In other words, to identify the individual at risk via a “*correlation matrix*” consisting of a *converter vs. a non-converter* system that indicates the population who might, for example, develop dementia.

Katrin Rauen points to an example of a matrix used in an ongoing group assessment, a study under development in Zurich that aims to implement matrix analysis and interventional and clinical trials in terms of anti-tau and anti-inflammatory compounds [1,2].

In the context of the “correlation matrix”, Juan Carlos Arrago Lasprilla points to the fact that the question is whether TBI is a risk factor for developing dementia in the future as pre-clinical symptoms like fatigue, attention or cognition impairments are symptoms that the specialized literature presents as also pertaining to depression, so attention needs to be given to the overlaps that concur and might occur.

Volker Hömberg's intervention in the panel discussion underlines the necessity to think about what should be done to offer effective treatment solutions, *i.e*., to monitor the patients from a long-term perspective, identify the need for intervention and the type of reasonable, affordable, and necessary interventions and, to achieve this, a diagnostic scale becomes a requisite.

Marina Zeldovich, also supported by Nicole von Steinbüchel, presents a diagnostic scale that is under development in Germany, consisting of the following stages:

- Identify and access the problem;
- Provide a differential diagnosis, *i.e*., if it is TBI related or depression-related, or related to other causes;
- Treat the problem.

Still, in order to build a diagnostic scale, the two address the lack of a summary of all the findings in research, so it is very important to merge central findings from CENTER-TBI [3] and CAPTAIN studies, for example, in order to generate sensitive assessments, validate them and consolidate the results. Or, in Nicole von Steinbüchel's words, to identify sensitive instruments and validate them in order to treat TBI. The latter underlines the fact that there is no integrated information on neuropsychological data in observational studies – what kind of therapy is used, how often, and at what intensity, all of these remaining questions without a clear answer.

Nada Andelic states that there should definitely be developed follow-up programs [4,5], *e.g*., for community integration, and Johannes Vester contextualizes the entire problem as having financing as an important bottleneck, becoming thus a burden for society and the economy. Volker Hömberg states that a part of the problems could be resolved with digital technologies by developing applications [6,7] that could increase awareness by simultaneously generating data from the patients using a dedicated application. More than that, digital technologies are easily applicable, highly accessible, and trackable.

To support the idea launched by the previous panelist, Peter Lackner presents the work developed by a team he is also part of, namely an application for speech rehabilitation constructed on machine learning algorithms that can detect if the patient is progressive and, subsequently, assign heavier or easier tasks for the patient to deal with at home. Peter Lackner adds that the application is still in a study phase and is currently used only in hospitals under supervised learning.

However, such an application would work if machine learning algorithms would adapt to the difficulty of different tasks automatically and a central read-out would be obtained, thus placing all the results in a database, with the patients being remotely controlled.

Prof. Dafin F. Muresanu brings together under an innovative umbrella all opinions presented and brings forward the idea of a workgroup/task force, with representatives from and support of AMN, WFNR, EFNR, to be organized to work on various projects focused on developing digital products. Furthermore, NEUROTECH-^EU^ **–** The European University of Brain and Technology, would be extremely interested in benefiting from digital products developed in this medical field.
